# A Proposed Reform in Medical Education

**Published:** 1890-04

**Authors:** 


### A PROPOSED REFORM IN MEDICAL EDUCATION.

A SHORT time ago representatives of the faculties of the Baltimore Medical Colleges, held a meeting to consider how best to effect much needed reforms in medical education. As a result of the discussion on this occasion, they decided that the terms of study requisite for obtaining a diploma should be extended from two to three years, and that a national convention of medical schools should be formed in order to do away with the poor colleges, and still poorer students, who are entering the profession in great numbers each year. They decided to send out a circular to all the medical colleges in the United States, calling for a national conference. This circular briefly advocates concerted action on the part of all colleges in the country, believing that the schools of any state alone cannot expediently or practically assume alone the responsibility of adopting advanced methods. Faculties of other colleges are, therefore, invited to join in aiding this cause, by sending delegates to represent them, at a meeting, to be held at Nashville, Tenn., on the 21st day of May, 1890, at 3 P. M., at the time of the meeting of the American Medical Association. The following subjects are considered as most likely to come up for discussion:

1. Three years' course of six months' sessions.
2. Graded curriculum.
3. Written and oral examinations.
4. Preliminary examination in English.
5. Laboratory instruction in chemistry, histology and pathology.

We sincerely hope that the faculties of the various colleges may show their desire to effect these reforms, by sending well-instructed delegates to represent them, and that the conference of the same may lead to the adoption of such measures as are now urgently required. The degree of M. D. has almost as variable a significance to-day in this country as that of B. A., which we all know, may mean much or next to nothing at all. The country is becoming flooded with incompetent practitioners who impede the progress of the deserving, on account of the inability of the laity to discriminate between the scientific and the unscientific doctor, and who are constantly bringing our profession into disrepute.

In this connection it is of interest to note that the Faculty of Starling Medical College, of Columbus, O., has determined that three years of medical instruction shall be required by that institution of all candidates for the degree of Doctor of Medicine, after the season of 1890–91. A similar requirement has been imposed by the Faculty of the Hospital College of Medicine, Louisville, Ky.

While these gratifying indications of reform in medical education are appearing in the West, let us hope that the State of New York will take no step backward. If the effort to repeal the law of last year requiring a preliminary Regent's examination of all matriculates in medicine, should be successful (as we hear there is danger of), it would be a sad commentary on the intelligence of the legislators of the Empire State ; and, in that direful event, it would require much argument to convince the friends of medical educational reform that some persons in high places had not been "monkeying with the machine."

